# Structural and Electrical Studies for Birnessite-Type Materials Synthesized by Solid-State Reactions

**DOI:** 10.3390/nano9081156

**Published:** 2019-08-12

**Authors:** Nayda P. Arias, María E. Becerra, Oscar Giraldo

**Affiliations:** 1 Grupo de Investigación en Procesos Químicos, Catalíticos y Biotecnológicos, Universidad Nacional de Colombia-Sede Manizales, Kilometro 9 vía al aeropuerto, La Nubia, Manizales 170003, Colombia; 2 Facultad de Ciencias e Ingeniería, Universidad de Boyacá, Carrera 2ª Este No. 64–169, Tunja 15001, Boyacá, Colombia; 3 Laboratorio de Materiales Nanoestructurados y Funcionales, Facultad de Ciencias Exactas y Naturales, Universidad Nacional de Colombia-Sede Manizales, Kilometro 9 vía al aeropuerto, La Nubia, Manizales 170003, Colombia; 4 Departamento de Ingeniería Química, Facultad de Ingeniería y Arquitectura, Universidad Nacional de Colombia-Sede Manizales, Kilometro 9 vía al aeropuerto, La Nubia, Manizales 170003, Colombia; 5 Departamento de Física y Química, Facultad de Ciencias Exactas y Naturales, Universidad Nacional de Colombia-Sede Manizales, Kilometro 9 vía al aeropuerto, La Nubia, Manizales 170003, Colombia; 6 Departamento de Química, Universidad de Caldas, Calle 65 No 26-10, Manizales 17001, Colombia

**Keywords:** Birnessite, nanoporous metal oxides, impedance spectroscopy

## Abstract

The focus of this paper is centered on the thermal reduction of KMnO_4_ at controlled temperatures of 400 and 800 °C. The materials under study were characterized by atomic absorption spectroscopy, thermogravimetric analysis, average oxidation state of manganese, nitrogen adsorption–desorption, and impedance spectroscopy. The structural formulas, found as a result of these analyses, were $K_{0.29}^{+}\left(Mn_{0.84}^{4+}Mn_{0.16}^{3+}\right)O_{2.07}\cdot 0.61H_{2}O$ and $K_{0.48}^{+}\left(Mn_{0.64}^{4+}Mn_{0.36}^{3+}\right)O_{2.06}\cdot 0.50H_{2}O$. The N_2_ adsorption–desorption isotherms show the microporous and mesoporous nature of the structure. Structural analysis showed that synthesis temperature affects the crystal size and symmetry, varying their electrical properties. Impedance spectroscopy (IS) was used to measure the electrical properties of these materials. The measurements attained, as a result of IS, show that these materials have both electronic and ionic conductivity. The conductivity values obtained at 10 Hz were 4.1250 × 10^−6^ and 1.6870 × 10^−4^ Ω^−1^cm^−1^ for Mn4 at 298 and 423 K respectively. For Mn8, the conductivity values at this frequency were 3.7074 × 10^−7^ (298) and 3.9866 × 10^−5^ Ω^−1^cm^−1^ (423 K). The electrical behavior was associated with electron hopping at high frequencies, and protonic conduction and ionic movement of the K^+^ species, in the interlayer region at low frequencies.

## 1. Introduction

From the 1960s, works of Wolkestein [1] state the importance of electron theory to elucidate the relation between the catalytic and electronic properties of a catalyst and also the semiconductor nature of this type of material. Swaminathan [2] produced research about the opportunities and ways to improve catalyst technology, specifically in their development, cost reduction, and field applications [2]. Among the catalysts, semiconductors such as TiO_2_, ZnO, and SnO_2_ play a pivotal role, as they can use the electromagnetic spectra to degrade contaminants [3,4]. It has been reported that these semiconductor oxides also have acidic and basic natures [2]. Therefore, they are useful as both solid acid and base catalysts. For this reason, efforts to develop new, cheaper, active, and selective catalysts are ongoing. Manganese oxides are among the most extensively studied raw, supported, or doped catalysts [5,6,7,8,9,10,11]; however, their electrical properties have been less studied [12,13,14]. The family of the manganese oxide type of materials have variations in their structure depending on the way the Mn–O is linked together [15]. Therefore, layer, tunnel, spinel, and compact structures can be found. Manganese oxides have also been used as a cathode in secondary batteries [16,17]. Manganese, as a central atom in the octahedral coordination in these structures, mainly has the 4+ and 3+ oxidation states. However, average oxidations states of 3.5 and 3.8 are commonly found because of the presence of Mn^4+^ and Mn^3+^ in the same building blocks [18]. Birnessite is a special structure of this manganese oxide family. It is composed of Mn–O octahedra forming octahedral layers as clays, and it has monovalent or divalent ions surrounded by water molecules to compensate the electrical charge of its layers. Therefore, these monovalent or divalent cations are located in the region called the interlayer [9,12]. The cations in the interlayer region can be displaced by other species because of their mobile nature. Birnessite has been used in previous works as a catalyst in soot combustion processes and in methylene blue degradation, showing appreciable catalytic activity compared to traditional catalysts [9,11,19]. For this reason, the present work focuses on the charge transport mechanism for two birnessite material types, Mn4 and Mn8, synthesized at 400 and 800 °C, respectively, with the intention for a deeper understanding of the nature of this type of material for advanced applications.

## 2. Results and Discussion

### 2.1. Chemical Composition, Thermogravimetric Analysis (TGA), and Average Oxidation State (AOS)

The thermal reduction of KMnO_4_ has been reported by Kappestein [20] and Herbstein [21]. In the study conducted by Herbstein et al. [21] on the thermal decomposition of KMnO_4_ in a temperature range of 25 to 900 °C, in an atmosphere of air and nitrogen, it was found that the idealized equation for the decomposition of KMnO_4_ in air at 250 °C results in a soluble phase, the K_2_MnO_4_, and another phase that is insoluble, like this:

$$10\;KMnO_{4\;\underset{}{\to }}2.65\;K_{2}MnO_{4}+\left[2.35K_{2}O\;7.75MnO_{2.05}\right]+6O_{2}.$$

Decomposition in air or nitrogen at higher temperatures (up to 540 °C) results in more considerable amounts of O_2_, corresponding to the change in the composition of the nonsoluble phase. At temperatures above 540 °C in both air and nitrogen, K_2_MnO_4_ decomposes into:

$$10\;K_{2}MnO_{4\;\underset{}{\to }}5.7K_{3}MnO_{4}+0.5\left[2.9K_{2}O\;8.6MnO_{2.1}\right]+3.4O_{2}.$$

These potassium manganates are stable within the temperature range of 25 to 900 °C, but they react quickly with water vapor. The thermal decomposition of KMnO_4_ is a redox reaction in which the oxoanion oxygen is oxidized to molecular oxygen, while the oxidation number of manganese in the oxoanion is reduced from Mn (VII) to Mn (VI), from $MnO_{4}^{-}$ and $MnO_{4}^{2-}$. It has been reported that in the thermal decomposition of KMnO_4_ at 250 °C, an average of 1.6 Mn–O bonds are broken in 73% of the permanganate ions. The electrons are then transferred to the remaining 27% of permanganate ions, which are reduced to $MnO_{4}^{2-}$ without presenting a significant change in its tetrahedral form. In the K_2_MnO_4_ at 600 °C, 1.6 Mn–O bonds are broken on average in 43% of $MnO_{4}^{2-}$ ions, and the electrons are transferred to the remaining 57% of manganate ions, which are reduced to $MnO_{4}^{3-}$.

The manganates formed by this reaction are soluble salts. For this reason, the preparation of birnessite-type materials for this synthesis route involves a washing procedure after the calcination process to provide birnessite as the only crystallographic phase.

The K/Mn ratio, obtained by atomic absorption (AA) results (Table 1), shows a variation in the potassium and manganese content of the materials as the synthesis temperature increases [9].

**Figure 1:**
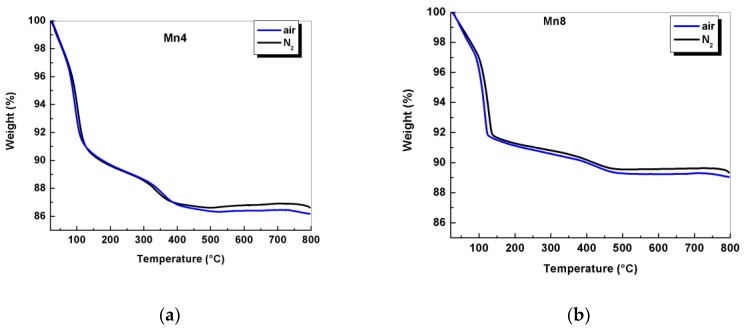
Thermograms in air and N_2_ for synthesized birnessite-type materials. (**a**) birnessite type of material synthezised at 400°C, (**b**) birnessite type of material synthezised at 800°C.

Similarly, a decrease in the average oxidation state (AOS) (Table 1) indicates the presence of manganese in the 4+ and 3+ oxidation states. The percentage of Mn^3+^ in these materials was 16%, and 36% for Mn4 and Mn8, respectively. The results of the AOS, for Mn4 and Mn8, are close to those reported in the literature [9,22] for birnessite produced by solid-state reactions at 400 and 800 °C. Thermal analysis in nitrogen and air atmospheres (Figure 1) showed thermal events typical of these layer materials [9,22,23]. Weight loss, up to 250 °C, is attributed to the evaporation of physiosorbed water and interlayer water. From 250 to about 800 °C, the materials lose oxygen because of the phase transformation from a layer to a tunnel structure [9]. When heated to temperatures above 250 °C, the Mn4 material loses 2.48% of its original mass in nitrogen atmosphere. Similarly, Mn8 experienced a weight loss of 1.69%. These results indicate that the materials synthesized at higher temperatures lose less oxygen, and show a greater thermal stability, compared to the ones synthesized by chemical routes [24]. The materials were more stable in nitrogen than in air, as it can be seen in Figure 1. The difference in weight losses at temperatures higher than 250 °C between both atmospheres indicates a release of oxygen from the framework, as it was reported by Suib et al. [25]. For Mn4, this difference was 0.53%, and it was 0.16% for Mn8. The combined results of atomic absorption, thermogravimetric analysis (TGA), and AOS were used to formulate the structural formula shown in Table 1.

### 2.2. Structural Analysis and Rietveld Refinement

**Figure 2:**
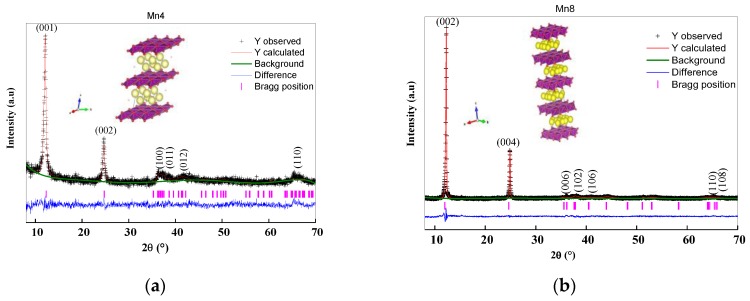
Rietveld refinement results for (**a**) Mn4, and (**b**) Mn8. The insert shows the polyhedral structure of the triclinic and hexagonal birnessite for (**a**) Mn4, and (**b**) Mn8 respectively.

The synthesis method produced a typical layered manganese oxide (birnessite) structure, as confirmed by X-ray diffraction (XRD) (Figure 2a). The d (001) basal spacing and estimated crystal size are shown in Table 1. The basal spacing of these layer materials is in accordance with the published works [18,22,26,27].

Analysis between 30° and 70° in 2θ showed the presence of structural disorders associated with the rotation and translational stacking faults of the layers, as demonstrated in phyllosilicate minerals [28]. The synthesis temperature and structural disorder ratio was also noted by Gaillot et al. [22,29]. The Mn4 material (Figure 2a), in the range mentioned above, had diffraction peaks located at 2.91, 2.47, 2.40, 2.29, 2.17, 1.81, 1.43, 1.39, and 1.35 Å, which are in agreement with those reported for triclinic birnessite [29,30]. For Mn8 it was observed that X-rays diffracted at angles of 12.42°, 25.06°, 36.48°, 38.54°, 41.36°, 44.48°, 44.80°, and 53.46° 2θ, which correspond to interplanar spacing of 7.12, 3.55, 2.46, 2.33, 2.18, 2.03, 2.02, and 1.71 Å, respectively. These are characteristic of a hexagonal birnessite [29,30], an observation that also appears in Kim et al. [31].

The atomic position (fractional coordinates) can be shown in Appendix A, Table A1.

The structural symmetry of birnessite was confirmed by the Rietveld method. The structural parameters have been summarized in Table 2, and comparisons between the experimental and theoretical patterns are shown in Figure 2. The refined parameters confirm the triclinic structure for Mn4 and the hexagonal structure for Mn8 (Table 2). For Mn8 material, the crystallographic density value was higher than Mn4 (Table 2). The change in the crystallographic density for Mn8 was related to the variation in the crystal symmetry from triclinic to hexagonal. The c-cell parameter increased because of the rearrangement of the atoms in the interlayer region and the densification that resulted from temperature increase.

A typical Mn-O1 bond distance, in the layer of the analyzed materials, falls between 1.088–2.352 Å and 1.860 Å for Mn4 and Mn8 materials, respectively. The presence of Mn^3+^, in both the high- and low-spin states, as was evidenced by AOS (Table 1), was confirmed by the four short and two long Mn–O distances, which resulted from Jahn–Teller distortion. The Jahn–Teller distortion has important implications for the electrical conductivity of these materials. The Mn–O bond distances are in close agreement with those reported by Drits et al. [32], Gaillot et al. [22], Lopano et al. [33], and Post and Veblen et al. [34]. The crystal broadening, for Mn4, is isotropic, as can be seen in the aspect ratio index (Table 2), whereas it was anisotropic for Mn8. The crystal anisotropy affects the electrical properties, as will be discussed later.

### 2.3. Morphological Analysis

**Figure 3:**
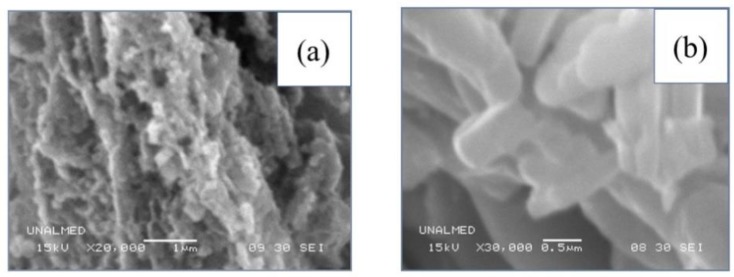
SEM images for Birnessite-type materials (**a**) Mn4 and (**b**) Mn8.

Scanning electron microscopy analysis (Figure 3) shows that Mn4 (Figure 3a) was formed by particle aggregates small in size compared to Mn8 (Figure 3b). When subjected to calcination at 800 °C (Mn8), these agglomerates grew uniformly. These agglomerates tended to have a hexagonal plate morphology and had smoother surfaces, which is characteristic of these types of materials. These observations are consistent with the crystal size/temperature increment relation showed by XRD (Figure 2, Table 1) and Rietveld refinement (Table 2).

### 2.4. N_2_ Adsorption–Desorption Analysis

**Figure 4:**
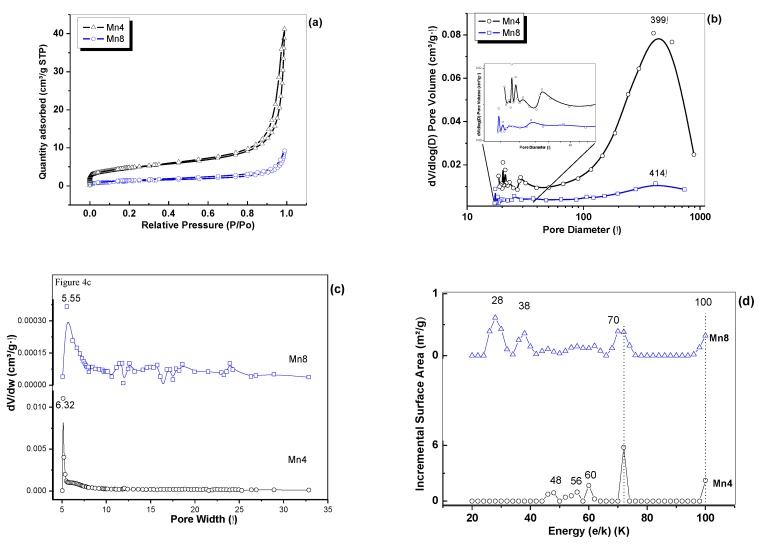
N_2_ adsorption–desorption results: (**a**) N_2_ isotherms, (**b**) mesopore size distribution by Barret–Joyner–Halenda (BJH), (**c**) micropore size distribution by Hovarth–Kawazoe (HK), and (**d**) interaction energy by nonlocal density functional theory (NLDFT).

The N_2_ adsorption–desorption isotherms of the materials (Figure 4a) showed the existence of a type II isotherm, according to the International Union of Pure and Applied Chemistry (IUPAC) classification [35] and H3 hysteresis, characteristic of materials with slit-shaped and large pores [36]. The surface area, found by using the Brunauer-Emmet-Teller (BET) method (Table 1), decreased as the synthesis temperature increased. This trend is consistent with the crystal growth–smoothness relation, as evidenced by XRD (Figure 2) and SEM (Figure 3). The mesopore size distribution (Figure 4b) showed peaks centered at around 399.3 and 414.7 Å for Mn4 and Mn8, respectively, with a pore volume that decreased as the synthesis temperature increased. This observation is in accordance with the aggregate size increment of the birnessite particles. All materials showed microporosity, as observed in Figure 4c, and the data show that the microporosity changes with increasing synthesis temperature. The interaction energy of the porous solid surface with the N_2_, obtained by nonlocal density functional theory (NLDFT) [37], varied with the synthesis temperature for the birnessite-type material. The data presented in Figure 4d show values for the interaction energy around 48, 56, 60, 72, and 100 K, with a greater contribution at 72 K for the Mn4 material. The main contribution for Mn8 occurred at 28 K followed by interaction energies of 38, 70, and 100 K. Maddox et al. [38] reported that in mesoporous materials, like MCM41, values below 75 K correspond to the usual range of values reported for O–N_2_ interactions. Values between 125 and 75 K correspond to theoretical εsf/k values for the O–O interaction [38]. Therefore, the information presented asserts that the surfaces of the birnessite-type material are energetically heterogeneous, which probably contributes, in a positive manner, to its electrical processes.

### 2.5. Electrical Analysis of Nyquist Plots

**Figure 5:**
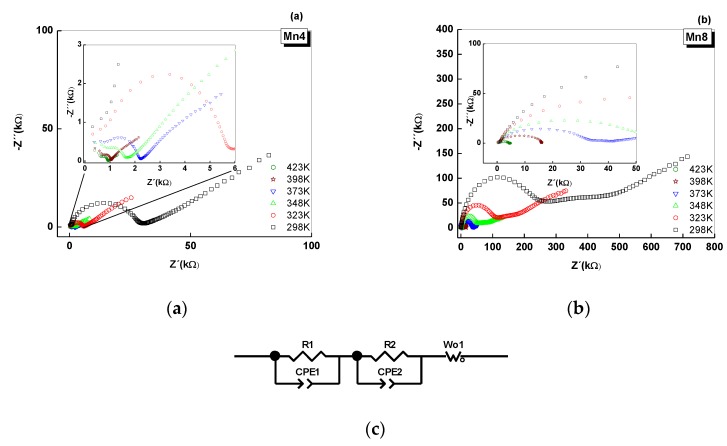
Nyquist Plot as a function of temperature for (**a**) Mn4 and (**b**) Mn8. (**c**) equivalent circuit. R1 and R2: resistances; CPE-1 and CPE-2: constant phase element; and Wo1: Warburg open element. The insert in (**a**) corresponds to an increase in the scale between 0 to 8 kΩ, The insert in (**b**) corresponds to an increase in the scale between 0 to 50 kΩ.

Nyquist plots from the impedance experimental data were conducted with the aim of studying the effect of temperature on the electrical response of the synthetized materials; the results are shown in Figure 5. The Nyquist plot exhibits a semicircle and a straight line at low frequency, but the magnitude of the diameter of the semicircle reduced as the temperature increased in both studied materials. This experimental evidence suggests an impedance reduction of the “bulk” and grain boundary processes. The presence of the semicircle in the Nyquist plot was associated with the electron transport processes on the “bulk”, in which there are a contribution of inter-crystalline and intra-crystalline charge transport processes. Also, in this region, a possible additional contribution to the electrical response came from protonic conduction (H_3_O^+^) because of the interlayer water in the birnessite structure. The observations corresponding to the electron transport process are concurrent with the results of previous research [12,13]. However, the preparation methods reported here produced materials with less impedance compared to previous work [12]. Therefore, is possible to tune the electrical properties of manganese oxides. The straight line at low frequency was associated with the ionic diffusion of K^+^ ions located in the interlayer region, which follow a Warburg diffusion process. The straight lines observed in Figure 5a,b indicated that the ionic diffusion was preserved up to 373 K, due to the satisfactory thermal stability of these materials (Figure 1), as opposed to soft synthesis routes [12,23].

The equivalent circuit, shown in Figure 5, that represents the experimental results, was constructed considering the morphology and structural issues for the studied materials, and it also takes into account the reports of the literature for the electrical behavior of porous materials [12,13,39,40,41,42]. In this circuit, each circuital element is composed of a constant phase element (CPE) connected in parallel with one resistor (R). CPE1 and R1 reflect the intra-/inter-crystalline electrical process, while CPE2 and R2 were assigned to fit the results concerning the charge transport in the grain boundary or aggregate boundary. These two circuital elements were put in series because it was the better circuit that better adjust the experimental results. Also, each of the circuital elements were connected in series because the Nyquist plot described consecutive processes [43], as reported in previous work [12]. The circuit was finally completed with a Warburg open element (W1). In these equivalent circuits, the constant phase elements (CPE1, CPE2) were selected based on SEM (Figure 3) and textural analysis (Figure 4). CPE describes a nonideal capacitive process, [12,44] which, for the purposes of this study, was used to model the porous structure of the material and its heterogeneous surface (Figure 4a–d) [45,46]. The numerical results of the equivalent circuits are summarized in Appendix A (Table A2 and Table A3).

In the circuital model, R1 and R2 are the inter-/intra-crystalline resistance and grain boundary resistance, respectively; CPE1-T is the capacitance of inter-/intra-crystalline processes; CPE2-T corresponds to the capacitance of the grain boundary process; and CPE1-P and CPE2-P are the exponents of the impedance for CPE (Equation (1)) for the inter-/intra-crystalline and grain boundary processes.

(1) $$Z_{CPE}=\frac{1}{\left(T{\left(I\times \omega \right)}^{P}\right)}$$

where $Z_{CPE}$ is the impedance of the CPE element, *T* corresponds to CPE-T, *P* corresponds to CPE-P, *ω* = angular frequency of alternating current signal, and $I=\sqrt{-1}$.

In the same model, Wo-R is the resistance to the ionic diffusion, and Wo-T and Wo-P are the coefficients according to the Warburg impedance equation (Equation (2)):

(2) $$Z_{Wo}=\frac{R\left(ctgh{\left(I\times T\times \omega \right)}^{P}\right)}{{\left(I\times T\times \omega \right)}^{P}},$$

where *Zw_o_* is the impedance for the Warburg open element, *R* describes the diffusion resistance (Ω), *P* is the exponential coefficient, and *T* is described through Equation (3):

(3) $$W_{0}-T=\frac{L^{2}}{D},$$

where *W*_0_ − *T* is the Warburg *T* coefficient, *L* is the effective diffusion thickness (m^2^), and *D* (m^2^s^−1^) is the effective diffusion coefficient of the charge carrier. The contribution of the charge transport processes, and their accumulation at the grain boundaries of the Birnessite particle’s aggregates, overlap with the “bulk” contribution because of the proximity of the particles, which was produced by the sample preparation for the electrical experiments (Figure 3). Because of this reason, the CPE2 and R2 elements were introduced into the circuital model.

The increase of the resistance of the “bulk” process (intra-crystalline and inter-crystalline), in the order Mn4 < Mn8 (Table A2 and Table A3, Appendix A), suggests an interplay between the crystal size, the average oxidation state of manganese, and the crystal symmetry (Table 1 and Table 2). Therefore, the smaller the crystal size is, the higher the micropore and surface area. For instance, Mn4, compared to the other cases, allows the electrons to travel through the crystal more freely because of the shorter Mn–O bond distances, as discussed above.

**Figure 6:**
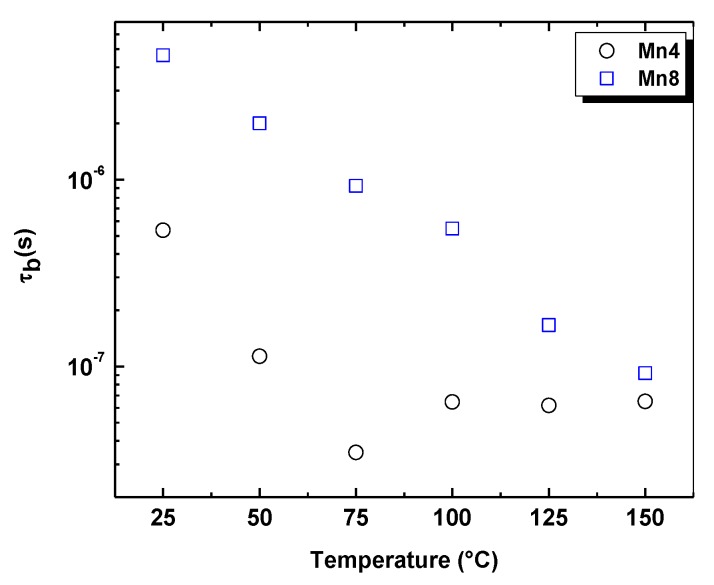
Effect of temperature on bulk relaxation time (τ_b_) for the synthesized birnessite-type material.

The values for the relaxation time are presented in Figure 6. The increase in relaxation time with temperature is consistent with the decrease of the resistance at “bulk process”, most likely due to higher K–O bond distances in Mn8, as was observed by the Rietveld refinement (Table 2). This relaxation time was almost linear with respect to the temperature for Mn8, but it decreased nearly exponentially for Mn4. This suggests that the relaxation time in the “bulk” is thermally activated and is lower for Mn4, indicating a fast electron mobility correlated to a high surface area and low crystal size (Table 1).

### 2.6. Alternant Current (AC) conductivity

**Figure 7:**
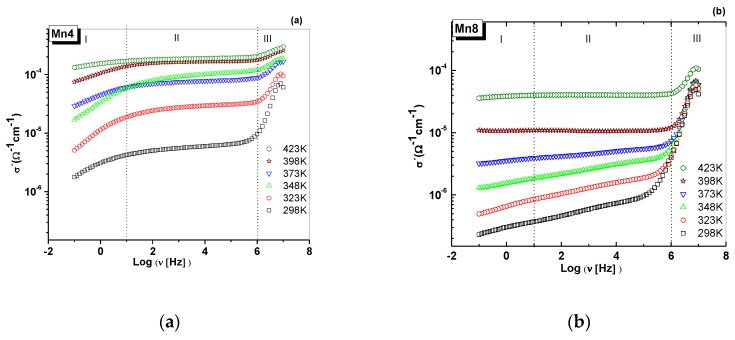
Real component of the complex conductivity as a function of temperature. (**a**) Mn4, birnessite type of material synthesized at 400°C, (**b**) Mn8, birnessite type of material synthesized at 400°C.

AC conductivity measurements (Figure 7) unraveled a power law behavior at high frequencies [47], which suggests electron hopping [48,49] and short-range conductivity [50]. The power law was first reported by Jonscher [47] and referred to as the universal dynamic response. It is described as

$$\sigma_{ac}=\sigma_{0}+A\omega^{n},$$

where *σ_ac_* is the AC conductivity, *σ*_0_ is the DC conductivity, *ω* is the angular frequency of the applied electric field, *ω* = 2πf, and *A* and *n* are the fitting parameters. *n* generally falls between zero and unity.

Such behavior has been observed in layered manganese oxides [12] and perovskite-type materials [51]. The sudden increment in the conductivity, above 10 Hz for Mn8 material (Figure 7), most likely is due to the high quantity of Mn^3+^ (Table 1) compared to Mn4. However, Mn4 had the highest conductivity (Figure 7) because of its smaller crystal size and higher surface area (Table 1). The conductivity values obtained at 10 Hz were 4.1250 × 10^−6^ and 1.6870 × 10^−4^ Ω^−1^cm^−1^ for Mn4 at 298 and 423 K, respectively. For Mn8, the conductivity values at this frequency were 3.7074 × 10^−7^ (298 K) and 3.9866 × 10^−5^ Ω^−1^cm^−1^ (423 K).

It is probable that the stacking faults in Mn4 provide crystallographic active sites for the movement of the charge carrier, as was evidenced in the surface energy plots (Figure 4c,d). Hence, it is probable that, at low frequencies, the ionic conduction process is the most favorable for Mn4, as represented by the appearance of the straight line in Figure 5a. Also, in this material, the K–O bond distances, the crystallographic position of K^+^, and the H_2_O interlayer allows the continuity in the channels for ionic conduction. Differences in the amount of potassium and water, and its position in the crystal cell of the studied materials, influence the electrical behavior in the low-frequency region. At higher contents of K^+^ ions, the two positions in the crystal cell, for the water molecules in Mn8, and the proper alignment of the material´s layers limit the ionic conduction routes, such that this contributes to a diminished ionic conductivity at low frequency (Figure 7). These types of materials are ionic exchangers [52]; therefore, the ions in the interlayer space are mobile. Water is an important key for the mobility of the ions in the interlayer space [12,53]. A low amount of water in the interlayer region can diminish the ionic mobility of the ions [53]. Furthermore, the water´s ability to facilitate ion conduction is dependent on the charge density of these ion [12,53,54]. Another reason for the enhanced conductivity is that the stacking faults alter the electrical conduction pathways. It is probable that this heterogeneous surface creates interactions between the electric field and the charge carrier. The variations in surface energies (Figure 4c,d) support the heterogeneity of the material surfaces.

**Figure 8:**
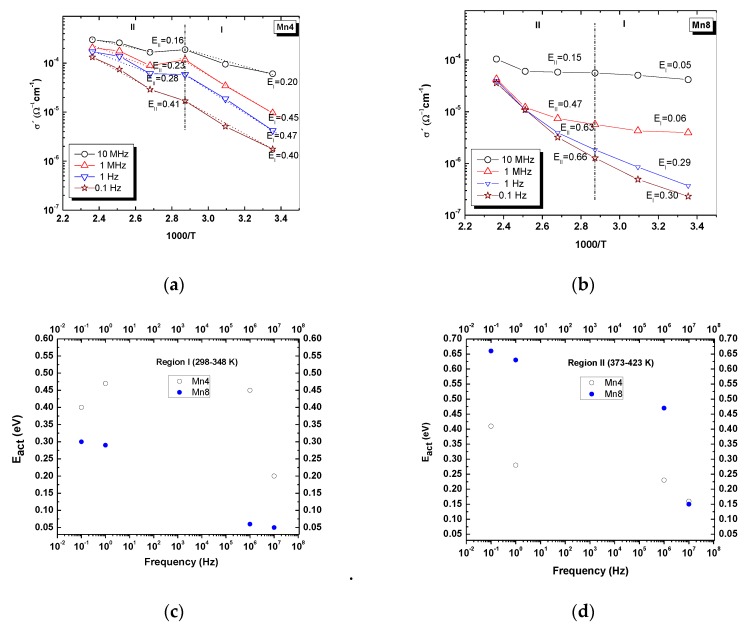
Arrhenius plot of the real component of the complex conductivity.(**a**) Mn4, (**b**) Mn8, (**c**) comparison of the activation energy between 298 to 348 K, (**d**) comparison of the activation energy between 373–423 K.

Figure 7 shows the AC conductivity for each material, by around two orders of magnitude, along with the induced temperature of the experiment. In Figure 7a, a low-frequency dispersion (LFD) can be seen for all temperatures. The region II (between 10 Hz up to 1 MHz) is dominated by DC conductivity, and region III (after 1 MHz) is characterized by the “universal Jonscher law” [47]. The results suggest that the charge transport mechanism is temperature-activated. Figure 8 shows the activation energy of each material. It is evident that, for Mn4 material (Figure 8a), the conductivity depends on the frequency and temperature. However, for Mn8 (Figure 8b), at temperatures higher than 348 K and frequencies below 10 MHz, the dependence is mainly associated with the temperature. The conductivity results suggest that the process is dominated by thermally activated “electron hopping” in the range of analyzed temperatures. When the electrons move forward between Mn sites, there is a change in the local structure caused by the change in the oxidation state of the MnO_6_ units that form the layer. These changes generate lattice vibrations. This phenomenon should be understood as a strong electron–phonon interaction or polaron mechanism [55], which is the other one present in the electrical conductivity process. The activation energies of each material (Figure 8) were lower than those reported for similar materials [12]. This is because of the higher average oxidation state of manganese (Table 1), which means a higher amount of Mn^4+^, as is reported for manganites obtained from a nickel permanganate precursor [56]. The lower activation energy of Mn4 compared to Mn6 and Mn8 could be associated with the difference in the quantity of Mn^3+^ between the synthesized materials, the variation of the Mn–O–Mn bond lengths resulting from crystal symmetry (Table 2), the changes of the surface area that can lead to a redistribution of electron charge [57], “polaron hopping” [58], and the Jahn–Teller distortion of Mn^3+^ ions [12]; thus, there are different electric behaviors with respect to the frequency of the applied electric field. Presumably, the increase of the conductivity involved in the transition zone, between Region I and Region II (Figure 8), was due to the release of physiosorbed water on the material’s surface [12].

The comparison of conductivity values for manganese oxides and birnessites synthesized in this work is shown in the Table 3.

## 3. Materials and Methods

### 3.1. Synthesis

Manganese oxides with birnessite-type structures (K-birnessite) were synthesized by the thermal reduction of a manganese salt, KMnO_4_ (Merck, Darmstand-Germany, 99%), as detailed in previous studies [9,22,31,67]. The procedure was as follows; the KMnO_4_ in powder form was homogeneously dispersed in a porcelain dish and exposed to calcination in a muffle furnace at temperatures of 400 and 800 °C for 6 h at 10 °C/min, to induce the thermal reduction, then they were cooled to room temperature. After calcination, each material was washed with deionized and distilled water to remove any soluble salts (potassium manganates) formed during the process and to reduce the pH from 12 to 9.5. The materials were dried at 60 °C for 24 h for further characterization. The materials were named as Mn4 and Mn8 (from thermal reduction at 400 and 800 °C, respectively).

### 3.2. Elemental Analysis and Average Oxidation State of Manganese

An elemental chemical analysis for the determination of K and Mn was performed by atomic absorption spectroscopy (AAS) in an atomic absorption spectrophotometer, PERKIN ELMER, model 3110, (Perkin Elmer Corporation, Waltham, MA, USA) and by using a standard procedure for sample digestion. The sample digestion process consisted of dissolving 100.0 mg of sample powder in 2.0 mL of 37% HCl and 1.0 mL of distilled and deionized water (DDW). The sample was then heated up to 100 °C, for about 30 min, until the solution became transparent and there were no undissolved solid particles. The Mn and K contents were determined at wavelengths of 279.5 and 766.5 nm, respectively. The average oxidation states (AOSs) of the manganese in the samples were determined by potentiometric and colorimetric titration [68]. In the first step, 40.0 mg of the sample was dissolved by heating in 18.5% v/v HCl solution, with the final volume being adjusted to 100 mL. After that, 10 mL of this solution was added to 100 mL of a saturated solution of Na_2_P_2_O_7_, the pH of the solution was adjusted to between 6.5–7.0, and then it was titrated with KMnO_4_ standard (around 1 × 10^−3^ M). The final point for this titration was taken when the potential increased to more than 100–150 mV. In the second step, about 40 mg of sample was dissolved in 10.0 mL of ferrous ammonium sulfate (FAS) solution under constant nitrogen flow, and the volume was adjusted up to 100 mL with DDW in order to reduce Mn^4+^ and Mn^3+^ to Mn^2+^. For the final step, 10.0 mL of this solution was back titrated, with a permanganate standard, until a color change occurred. All measurements were repeated three times in order to obtain the standard deviation.

### 3.3. X-ray Diffraction (XRD) Data Collection and Structural Refinement

All X-Ray Diffraction (XRD) patterns of the powder samples were performed at room temperature with a Brag–Brentano focusing geometry in a RIGAKU MINIFLEX II diffractometer, (Rigaku Company, Tokyo Japan), using CuKα radiation at 30 kV and 15 mA with a scan rate of 2° (2 θ min^−1^, sampling width of 0.01° (2θ), and between 3° and 70° (2θ). Rietveld refinement [69], for the obtained birnessite-type materials, was performed with a General Structure Analysis System (GSAS) [70] using the EXPGUI interface [71]. An experimental XRD pattern of a silicon powder was refined in order to obtain the instrumental parameter file. Because of the differences in the layer arrangements between each material, the initial structural parameters, triclinic [33] and hexagonal [22] symmetries, were used. The crystallographic information file (CIF) was obtained from Crystallography Open Database [72,73]. All polyhedral crystal structure drawings were made by using Vesta software.

### 3.4. Thermal Analysis

The thermal stability of the materials was studied by thermogravimetric analysis (TGA) on a TA instruments, model TGA Q500 thermogravimetric analyzer (TA Instrument, Delawere, DE, USA). Measurements were made on 10.0 mg of sample, using a high-resolution algorithm (sensitivity: 1, resolution: 5) with N_2_ flow (100 mL min^−1^) at a heating rate of 10 °C min^−1^. The measurement range was from 21 up to 800 °C.

### 3.5. Surface Area and Porosity

N_2_ adsorption–desorption isotherms were conducted to study the surface area, pore volume, and pore size distribution of the materials. The samples (100.0 mg) were degassed under vacuum at 120 °C for 24 h, and the adsorption–desorption isotherms were taken in a MICROMERITICS equipment model ASAP 2020, (Micromeritics, Norcross (Atlanta), Georgia, GA, USA), at 77 K with a pressure range from 1 × 10^−7^ P/Po to 0.99 P/Po. The specific area was calculated by the Brunauer–Emmett–Teller (BET) method. The Barret–Joyner–Halenda (BJH) method for mesopores and the Hovarth–Kawazoe (HK) method for micropores were used to determine the pore size distribution. To predict the surface energy of the synthesized materials, nonlocal density functional theory (NLDFT) was applied.

### 3.6. Scanning Electron Microscopy (SEM)

Micrographs were obtained in a JEOL JSM 5910LV microscope, (JEOL company Akishima, Tokyo, Japan), equipped with a secondary electron (SEI) detector operated at 15 kV in high-vacuum mode. For this analysis, around 3.0 mg of the synthesized material was placed onto a carbon ribbon, and then a thin layer of gold was deposited onto the surface of the sample by a sputter coating technique.

### 3.7. Impedance Spectroscopy Analysis

For impedance spectroscopy analysis, expressed as a function of temperature, the powder samples were uniaxially pressed at about 51 MPa to obtain pellets approximately 1.37 mm thick and 10 mm in diameter [12,14]. The pellets were kept between two platinum electrodes (the effective diameter for the working electrode was 10 mm) under spring-loaded pressure. The setup was put inside a furnace with temperature control from 298 to 423 K in steps of 25 K, and, in this case, measurements were taken using a dielectric interface SOLARTRON 1296 coupled to SOLARTRON 1260 analyzer (Solartron Analytical, Farnborough, UK). The cell was allowed to reach thermal stability for about 20 min before each measurement. Data were recorded in a frequency range of 10 MHz to 0.1 Hz with a voltage amplitude of 100 mVrms and 0 DC bias. The fitting circuit simulation of AC impedance data was performed with ZView^®^ (Scribner Association, Southern Pines, North Carolina, NC, USA) software.

## 4. Conclusions

Changes in symmetry and microstructure of synthesized birnessite-type materials produced variations in their electrical properties and relaxation times for the electrical transport mechanism. Mn4 exhibited the highest conductivity, which was associated with turbostratic disorder, shorter Mn–O bond length, lower crystal size, higher Mn^4+^ content, and higher surface area. For Mn8, the crystal size increment might have diminished the contact area between the particle agglomerates, making electron transport more difficult than in the Mn4 material. The enhanced conductivity with temperature suggests a thermally activated electron “hopping” and semiconductor behaviour for the synthesized materials. It is concluded that conductivity and other electronic-related phenomena can be tuned by varying the synthesis conditions.

## Figures and Tables

**Table 1:** Structural formulas for birnessite-type materials.

Material	Structural Formula	K/Mn	Average Oxidation State (AOS)	d_001_ (Å)	Crystal Size * (nm)	Specific Surface Area (m^2^/g)	Bulk Conductivity ** (Ω^−1^cm^−1^) × 10^−5^
**Mn4**	$K_{0.29}^{+}\left(Mn_{0.84}^{4+}Mn_{0.16}^{3+}\right)O_{2.07}\cdot 0.61H_{2}O$	0.29	3.84	7.12	25	16.26 ± 0.17	2.106
**Mn8**	$K_{0.48}^{+}\left(Mn_{0.64}^{4+}Mn_{0.36}^{3+}\right)O_{2.06}\cdot 0.50H_{2}O$	0.48	3.64	7.11	61	4.56 ± 0.04	2.135

* By Debye–Scherrer Equation, ** 21 °C.

**Table 2:** Summary of Rietveld refinement results for Mn4 and Mn8 materials.

Sample Parameter		Mn4	Mn8
Space group		P-1	P63/mmc
Data/Parameters		3074/26	3249/26
Mn1 (Occ)		1	1
K1 (Occ)		0.29	0.09
O1 (Occ)		1.02	0.95
O2 (Occ)		0.27	0.56
Mn1(Uiso)		0.005800	0.012370
K1 (Uiso)		0.067300	0.094000
O1 (Uiso)		0.031800	0.017300
O2 (Uiso)		0.067300	0.080000
Lattice parameters	a (Å)	2.91846 ± 0.005645	2.874708 ± 0.002132
	b (Å)	2.93104 ± 0.005117	2.874708 ± 0.002132
	c (Å)	7.43240 ± 0.006679	14.168819 ± 0.000357
	α (°)	78.2025 ± 0.184	90
	β (°)	103.5718 ± 0.166	90
	γ (°)	121.7815 ± 0.093	120
Volume (Å^3^)		52.284 ± 0.140	101.403 ± 0.123
Calculated Unit Cell Molecular Weight		108.114	214.884
Crystallographic density (g/cm^3^)		3.434	3.531
Perpendicular crystal size (nm)		18	53
Parallel crystal size (nm)		15	198
Aspect ratio		0.83	3.74
χ^2^		0.9317	1.460
R_p_		0.0209	0.0224
R_wp_		0.0266	0.0293
R_f_		0.4121	0.0315
R_exp_		0.0276	0.0242

Mn1: Mn layer, O1: O layer, and O2: O from interlayer water.

**Table 3:** Conductivity values for different manganese oxides.

Phase	Conditions	Conductivity	Reference
MnO	Room temperature	10^−9^ Ω^−1^cm^−1^	[59]
birnessite	298 K	10^−5^to 10−^6^ Ω^−1^cm^−1^	[60]
Todorokite	298 K	2.083 × 10^−6^ Ω^−1^cm^−1^ (4.8 × 10^5^ Ωcm)	[60]
MnO_2_	Room temperature	Resistivity 0.5 ohm-cm (2 Ω^−1^cm^−1^)	[61]
Mn_2_O_3_	Room temperature	Resistivity 0.0028 ohm-cm	
Mn_2_O_3_	Room temperature Under pressure 43 GPa	~10^−3^ Ω^−1^m^−1^	[62]
MnO_2_	Room temperature	Resistivity 6.8 × 10^−7^ Ωm	[63]
B-MnO_2_	Room temperature	1.27 × 10^−4^ Ω^−1^cm^−1^ 3.18 × 10^−5^ Ω^−1^cm^−1^	[64]
Mn_3_O_4_	323 K	(3.68 ± 0.03) × 10^−8^ Ω^−1^m^−1^	[65]
Mn_3_O_4_	423 K	(2.04 ± 0.001) × 10^−5^ Ω^−1^m^−1^	[65]
MnO_2_ todorokite		4.9 × 10^−2^ Ω^−1^cm^−1^ for nanowires	[66]
MnO_2_ criptomelane		10^−3^ to 10^−4^ Ω^−1^cm^−1^	[15]
MnO_2_ criptomelane		10^−6^ Ω^−1^cm^−1^	[52]
K-birnessite		10^−6^ Ω^−1^cm^−1^	
Na Birnessite	298 K, 1 MHz	8.39 × 10^−6^ Ω^−1^ cm^−1^	[12]
K-birnessite	298 K, 10 Hz	Mn4: 4.1250 × 10^−6^ Ω^−1^cm^−1^, Mn8: 3.7074 × 10^−7^ Ω^−1^cm^−1^	This Work
	298 K, 1 MHz	Mn4: 6.555 × 10^−5^ Ω^−1^cm^−1^ Mn8: 3.944 × 10^−6^ Ω^−1^cm^−1^	This Work
	423 K, 10 Hz	Mn4: 1.6870 × 10^−4^ Ω^−1^cm^−1^ Mn8: 3.85 × 10^−5^ Ω^−1^cm^−1^	This Work
	423 K, 1 MHz	Mn4: 2.050 × 10^−4^ Ω^−1^cm^−1^ Mn8: 4.270 × 10^−5^ Ω^−1^cm^−1^	This Work

## Appendix A

**Table A1:** Fractional coordinates for atom positions in Mn4 and Mn8.

		Mn4	Mn8
Fractional Coordinates Mn1	x	0	0
	y	0	0
	z	0	0
Fractional Coordinates K1	x	1.625160	0.127491
	y	0.835220	0.872509
	z	0.407410	0.250000
Fractional Coordinates O1	x	0.223160	0.666700
	y	0.821360	0.333300
	z	0.094050	0.059257
Fractional Coordinates O2	x	0.032800	0.333300
	y	−0.059160	0.666700
	z	0.554910	0.250000

**Table A2:** Circuital element values from equivalent circuit fitting of the experimental results for Mn4.

T (K)	CPE1-T (F)	CPE1-P	Rg (Ω)	CPE2-T (F)	CPE2-P	Rgb (Ω)	Wo-R	Wo-T	WoP
298	1.40 × 10^−11^	1.04	1.67 × 10^5^	3.18 × 10^−10^	0.94	8.95 × 10^3^	3.05 × 10^4^	3.45 × 10^−1^	0.22
323	5.54 × 10^−11^	0.95	4.85 × 10^4^	6.23 × 10^−12^	0.12	3.02 × 10^2^	3.98 × 10^3^	2.86 × 10^−2^	0.22
348	1.58 × 10^−9^	0.76	1.30 × 10^3^	6.18 × 10^−19^	0.94	157 × 10^2^	8.63 × 10^2^	5.41 × 10^−3^	0.20
373	3.06 × 10^−9^	0.70	2.13 × 10^3^	2.09 × 10^−17^	2.00	1.11 × 10^1^	1.22 × 10^2^	8.23 × 10^−5^	0.17
398	9.65 × 10^−10^	0.78	8.84 × 10^2^	1.00 × 10^−20^	2.91	1.28 × 10^2^	1.04 × 10^−1^	1.49 × 10^−12^	0.17
423	2.08 × 10^−9^	0.77	6.74 × 10^2^	1.00 × 10^−20^	2.92	1.03 × 10^2^	8.45 × 10^−2^	5.15 × 10^−14^	0.12

CPE1-T and CPE1-P are the coefficients of the constant phase element in Equation (1); Wo-R, Wo-T, and WoP are the coefficients for Warburg open element in Equation (2); and Rg and Rgb are the resistances in grain and grain boundary, respectively.

**Table A3:** Circuital element values from equivalent circuit fitting of the experimental results for Mn8.

T (K)	CPE1-T (F)	CPE1-P	Rg (Ω)	CPE2-T (F)	CPE2-P	Rgb (Ω)	Wo-R (Ω)	Wo-T (s)	WoP
298	2.51 × 10^−11^	0.98	2.08 × 10^5^	4.61 × 10^−8^	0.634	1.98 × 10^5^	1.07 × 10^6^	39.38	0.34
323	1.67 × 10^−11^	1.00	8.87 × 10^4^	8.56 × 10^−8^	0.618	6.75 × 10^4^	6.13 × 10^5^	45.61	0.32
348	1.37 × 10^−11^	1.02	4.34 × 10^4^	2.01 × 10^−7^	0.572	3.09 × 10^4^	2.10 × 10^5^	126.6	0.26
373	1.13 × 10^−11^	1.04	2.68 × 10^4^	6.93 × 10^−8^	0.680	8.53 × 10^3^	4.37 × 10^4^	91.41	0.19
398	2.10 × 10^−11^	1.00	8.29 × 10^3^	6.24 × 10^−4^	0.068	1.50 × 10^17^	N.A	N.A	N.A
423	4.36 × 10^−11^	0.97	3.69 × 10^3^	1.19 × 10^−3^	0.042	5.00 × 10^7^	N.A	N.A	N.A

CPE1-T and CPE1-P are the coefficients of the constant phase element in Equation (1); Wo-R, Wo-T, and WoP are the coefficients for Warburg open element in Equation (2); and Rg and Rgb are the resistances in grain and grain boundary, respectively.
